# Enhanced in vitro osteogenic differentiation of human fetal MSCs attached to 3D microcarriers versus harvested from 2D monolayers

**DOI:** 10.1186/s12896-015-0219-8

**Published:** 2015-10-31

**Authors:** Asha Shekaran, Eileen Sim, Kah Yong Tan, Jerry Kok Yen Chan, Mahesh Choolani, Shaul Reuveny, Steve Oh

**Affiliations:** Stem Cell Group, Bioprocessing Technology Institute, Agency for Science, Technology and Research (A*STAR), 20 Biopolis Way, #06-01 Centros, Singapore, 138668 Singapore; Department of Obstetrics and Gynaecology, Yong Loo Lin School of Medicine, National University of Singapore, Singapore, 119228 Singapore; Cancer & Stem Cell Biology Program, Duke-NUS Graduate Medical School, Singapore, 169857 Singapore; Department of Reproductive Medicine, KK Women’s and Children’s Hospital, Singapore, 229899 Singapore

**Keywords:** Stem cells, Microcarriers, MSC, Cell harvest, Osteogenic differentiation

## Abstract

**Background:**

Mesenchymal stem cells (MSCs) are of great interest in bone regenerative medicine due to their osteogenic potential and trophic effects. However, challenges to large-scale production of MSCs can hinder the translation of MSC therapies. 3D Microcarrier (MC)-based MSC culture presents a scalable and cost-effective alternative to conventional methods of expansion in 2D monolayers. Furthermore, biodegradable MCs may allow for MC-bound MSC delivery without enzymatic harvest for selected applications such as bone healing. However, the effects of cell expansion on microcarriers and enzymatic cell harvest on MSC phenotype and osteogenic differential potential are not well understood. In this study, we characterized human fetal MSCs (hfMSCs) after expansion in 3D microcarrier spinner or 2D monolayer cultures. Following expansion, we compared osteogenic differentiation of cultures seeded with 3D MC-harvested, 3D MC-bound and conventional 2D monolayer (MNL)-harvested cells when cultured in osteogenic induction media on collagen-coated plates.

**Results:**

Fetal MSCs expanded on both 3D agitated Microcarriers (MC) and 2D Plastic static monolayer (MNL) cultures express high levels of MSC surface markers. MC-harvested hfMSCs displayed higher expression of early osteogenic genes but slower mineralization kinetics compared to MNL-harvested MSCs during osteogenic induction. However, in the comparison between MC-bound and MC-harvested hfMSCs, osteogenic genes were upregulated and mineralization kinetics was accelerated in the former condition. Importantly, 3D MC-bound hfMSCs expressed higher levels of osteogenic genes and displayed either higher or equivalent levels of mineralization, depending on the cell line, compared to the classical monolayer cultures use in the literature (MNL-harvested hfMSCs).

**Conclusion:**

Beyond the processing and scalability advantages of the microcarrier culture, hfMSCs attached to MCs undergo robust osteogenic differentiation and mineralization compared to enzymatically harvested cells. Thus biodegradable/biocompatible MCs which can potentially be used for cell expansion as well as a scaffold for direct in vivo delivery of cells may have advantages over the current methods of monolayer-expansion and delivery post-harvest for bone regeneration applications.

**Electronic supplementary material:**

The online version of this article (doi:10.1186/s12896-015-0219-8) contains supplementary material, which is available to authorized users.

## Background

Mesenchymal stem cells (MSCs) are cells with the potential to differentiate into multiple cell types including osteoblasts, chondrocytes and adipocytes, and were shown to have trophic effects, modulate immune responses and promote healing in vivo [1]. In particular, the application of MSCs for bone repair has shown clinical promise and there is continued research interest in this area [2]. Human fetal bone marrow derived MSCs (hfMSCs) may be especially well suited for bone healing applications, as they can be maintained for many passages, have a faster doubling and greater osteogenic capacity compared to MSCs from other sources, such as human umbilical cord-derived and human adipose tissue-derived MSCs, and support in vivo bone formation [3–5].

Despite the promise of MSC-based therapies, MSC incidence in donor tissue is low [6, 7], and the estimated dosages required for clinical efficacy are high, ranging from 40 to 100 million cells per patient [8]. Therefore, the commercial viability and clinical translation of MSC therapies, especially those using allogeneic off-the shelf strategies, are hindered by challenges of scalable expansion of these cells [8]. Current methods of MSC expansion and production such as using stacked 2D surfaces as cell attachment substrates have limited scalability, are labor intensive and are costly when large numbers of cells are required [9].

One potential method of scalable expansion of MSCs involves the use of bioreactors for suspension culture and the use of 150–200 μm beaded microparticles, referred to as microcarriers, as cell adhesion supports. Recent research indicates that microcarrier/bioreactor systems which are currently primarily used for vaccine production may be adapted for MSC culture and may provide several benefits over current culture methods. Firstly, 3D microcarrier-based systems are more scalable than 2D monolayer culture systems, allow for ease of cell sampling and monitoring, and are efficient in terms of space and culture media usage [8]. Previous work in our lab has demonstrated efficient growth and differentiation of MSCs expanded on microcarriers in agitation culture systems [10]. Secondly, in addition to serving as support substrates for cell expansion, biodegradable 3D microcarriers [11–18] may potentially serve as scaffolds for in vivo MSC delivery for selected applications such as bone healing, eliminating the need for enzymatic harvest of MSCs from 3D microcarriers. This would require the development of biodegradable/biocompatible microcarriers.

In recent years there has been an increased research interest in the expansion and differentiation of MSCs or osteoblast-like cells on various naturally-derived, synthetic and hybrid 3D microcarriers such as PLGA [11, 12] ENREF_9, PEG [13], PCL [14], PLA [15], gelatin [16–18] ENREF 11 ENREF 11 ENREF 11 ENREF 11, collagen-coated polystyrene [19], gelatin-coated or uncoated dextran [10, 16, 20–22] ENREF 17 ENREF 17, charged cellulose [16], decellularized adipose tissue [23] and calcium phosphates [24, 25]. Microcarrier-expanded MSCs or microcarrier-laden MSCs have been combined with other strategies such as growth factor delivery [11], bioprinting [15], sintering [12], cell-microcarrier aggregation and perfusion [17] or incorporation into scaffolds or hydrogels [10] to enhance the cells’ regenerative potential, facilitate in vivo delivery or fabricate larger tissue constructs. Collectively, these studies have demonstrated that efficient expansion and differentiation of MSCs or osteoblast-like cells on microcarriers is feasible.

However, while much is known about the growth [16, 26, 27] of MSCs on microcarriers and to a lesser extent, on the mineralization potential of microcarrier-expanded cells, fewer studies have compared microcarrier-expanded cells, both harvested and unharvested, with conventional 2D monolayer harvested cells [28, 29] ENREF 26 to determine their phenotype and osteogenic differentiation potency. In this study, we expanded human fetal MSCs (hfMSCs) on 3D commercially-available Cytodex 3 gelatin-coated dextran microcarriers in spinner flasks or as a control, on 2D tissue culture plastic and gelatin-coated monolayer surfaces and characterized them. Following the expansion, we seeded and differentiated 3D Microcarrier-bound (3D MC-bound), 3D Microcarrier-harvested (3D MC-harv) and 2D Monolayer-harvested (2D MNL-harv) hfMSCs on collagen-coated plates or polycaprolactone -tricalcium phosphate scaffolds and investigated mineralization as well as the expression levels of key osteogenic genes under these conditions. 2D gelatin monolayer-harvested (2D gelatin-MNL-harv) human fetal MSCs on collagen-coated plates were also included as controls for the effects of gelatin on differentiation.

## Results

**Fig. 1:**
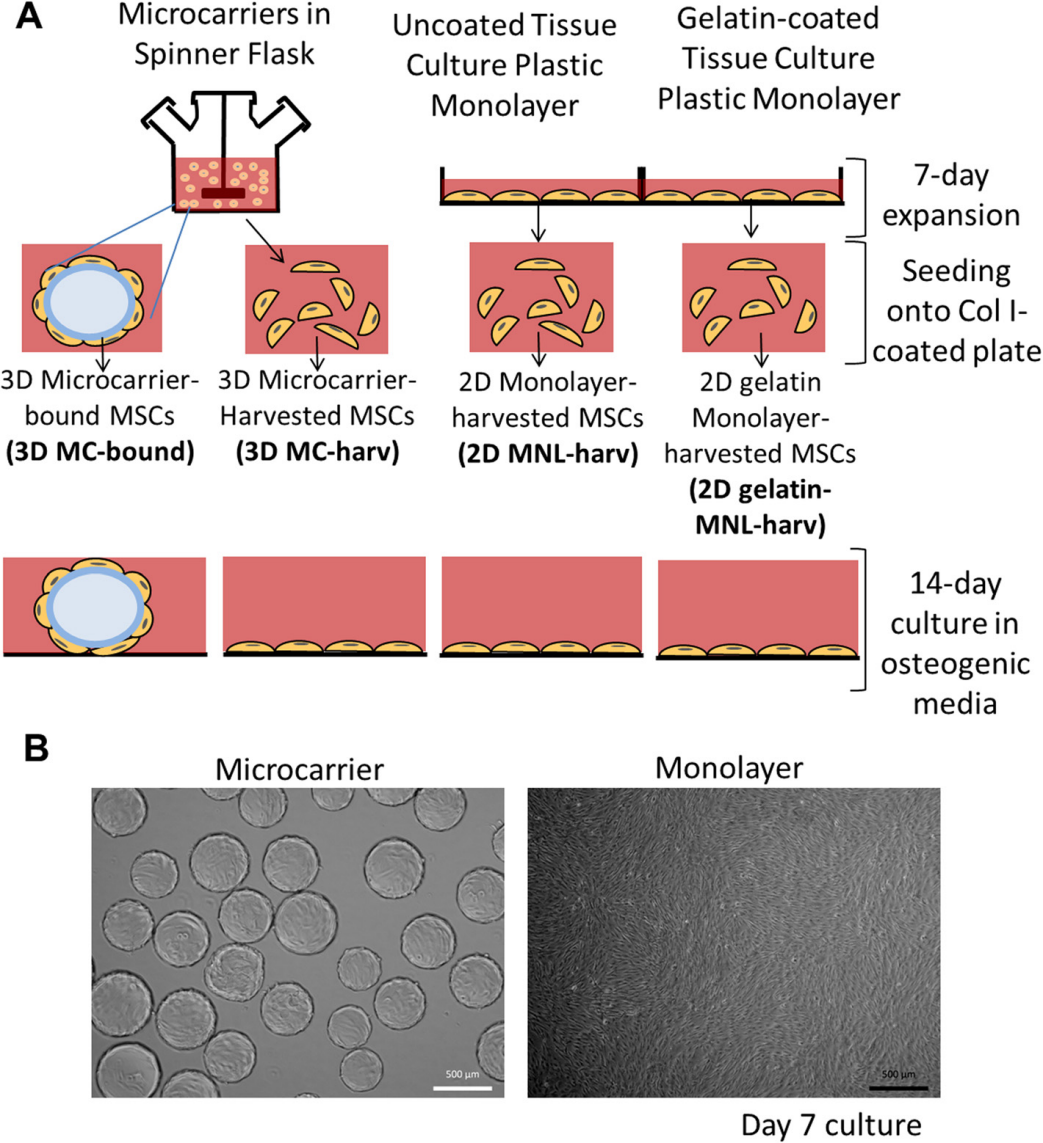
**a** Experimental Design. Human fetal mesenchymal stem cells (hfMSCs) from 2 different donors, denoted as S27 and S127 were cultured in growth media on Cytodex 3 microcarriers (3D MCs) in a spinner flask or as monolayers on tissue culture plastic flasks for 7 days. Thereafter, cells on 3D MCs (3D MC-bound), or cells enzymatically harvested from MCs (3D MC-harv) were seeded onto a collagen I-coated plate and differentiated for 14 days. Similarly cells expanded on uncoated (2D MNL-harv) or gelatin-coated (2D gelatin-MNL-harv) monolayer flasks were harvested and differentiated for 14 days. **b** Microscopic images of S27 hfMSCs after 7 days of expansion on Cytodex 3 microcarriers

To investigate the effect of microcarrier culturing condition and enzymatic cell harvesting on hfMSC phenotype after cell expansion and during osteoblastic differentiation, we expanded hfMSCs on microcarriers and monolayer cultures and osteogenically differentiated them with or without cell harvesting (Fig. 1a).

### Characterization of hfMSC expanded in 2D static monolayer and agitated 3D MC cultures

**Fig. 2:**
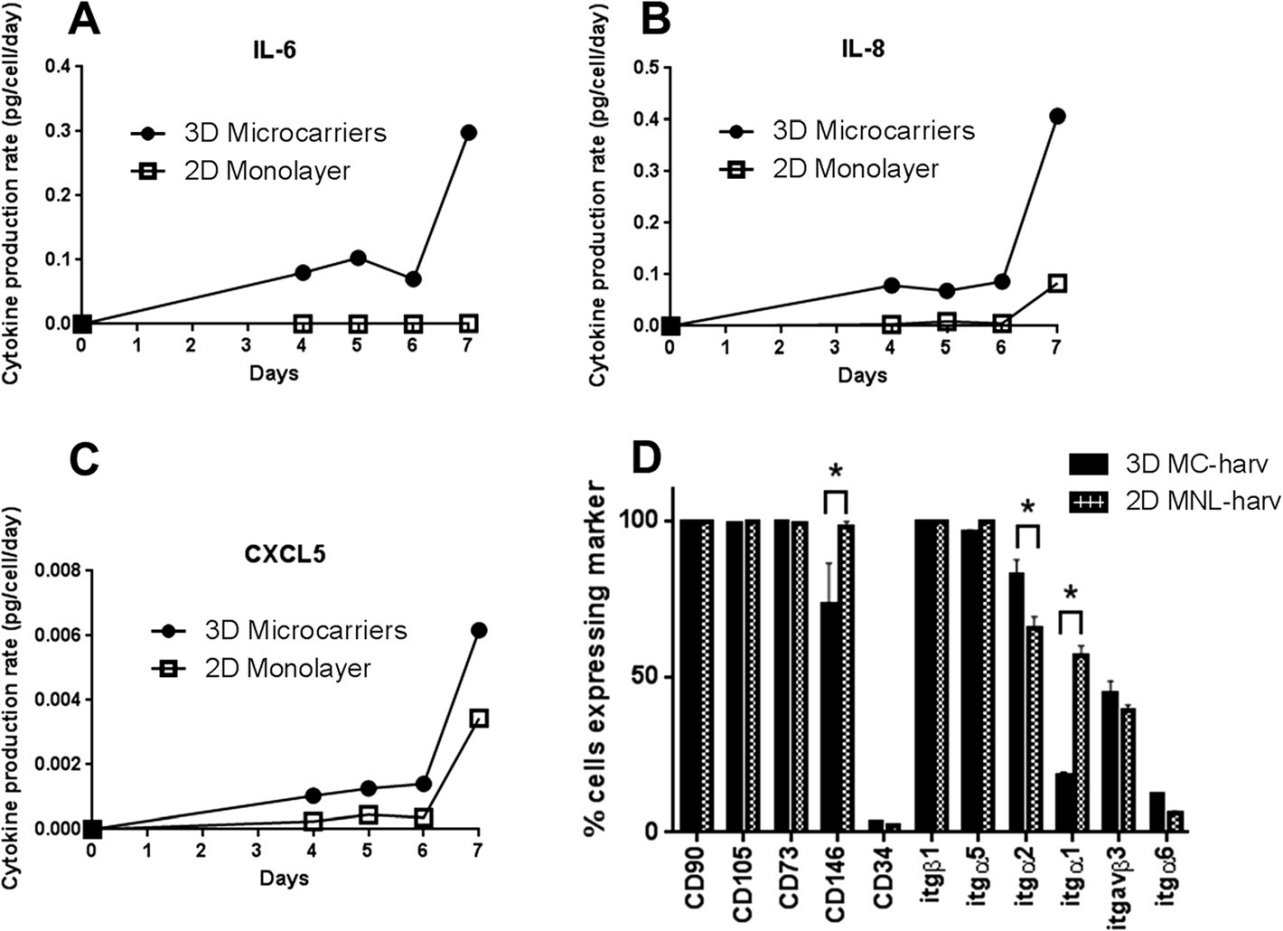
Phenotype of S27 hfMSCs expanded on 2D MNL or on Cytodex 3 microcarriers (3D MC) in a spinner flask. Profile of cytokine production rates for (**a**) IL-6, (**b**) IL-8 and (**c**) CXCL5 as measured by ELISA during the 7-day expansion. **d** Cell surface marker expression of MSC harvested from 2D MNL and 3D MC expanded cultures measured by flow cytometry. *p < 0.05 for 3D MC-harv compared to 2D MNL-harv by 2-tailed *t*-test in Graphpad

hfMSCs were expanded on microcarriers in spinner flasks or 2D plastic flasks in monolayer cultures. Cell seeding density was 4,444 cells/cm^2^ for microcarriers (4 cells/bead) and 1,142 cells/cm^2^ for plastic monolayers respectively to ensure that both cultures reached confluence after 7 days of expansion. Doubling times were 53.4 and 35.1 h for microcarrier and monolayer cultures respectively. At cell harvest after 7 days of expansion, the cell densities of hfMSCs were similar for microcarriers and plastic monolayer (39,352 cells/cm^2^ and 31,429 cells/cm^2^ respectively). At Day 7 of growth, the hfMSCs grew as monolayers in both MC cultures and 2D flasks, with no aggregation of MC cultures observed (Fig. 1b). Cytokines were secreted in 3D MC cultures at higher specific production rates compared to 2D monolayer cultures. At Day 7 of expansion, the specific production rates for 3D MC cultures over 2D monolayer cultures were 249 fold (0.2985 vs 0.0012 pg/cell/day) for IL-6, 4.9-fold (0.4072 vs 0.0836 pg/cell/day) for IL-8 and 1.8 fold (0.00618 vs 0.00344 pg/cell/day) for CXCL5 (Fig. 2a, b and c respectively).

Flow cytometry analysis of cell surface marker expression showed that 3D MC-harv hfMSCs expressed equivalent high levels of International Society for Cell Therapy (ISCT) MSC markers (CD34, CD105, CD73 and CD90) and lower levels of the endothelial, pericyte-associated marker CD146^27,28^ and α1 integrin (itgα1) and higher level of the pro-osteogenic α2 integrin (itgα2), compared to 2D MNL-harv hfMSCs (Fig. 2d). FACS data for cell surface marker expression was not obtained on 3D MC-bound cells because FACS analysis requires dissociating cells from the microcarriers. qPCR confirmed that the α2 integrin is upregulated in 3D MC-harv hfMSCs compared to 2D MNL-harv hfMSCs (Additional file 1: Figure S4).

In summary microcarrier culture increases the cytokine production rate and changes the expression levels of some cell surface markers, but not the ISCT MSC markers.

### Effect of hfMSC expansion methods on osteogenic differentiation (3D MC-harvested vs 2D MNL-harvested cells)

To determine the effect of 3D microcarrier-based cell expansion on subsequent osteogenic differentiation, we measured gene expression and calcium deposition during osteogenic differentiation on collagen-coated 6-well plates seeded with 3D MC-harvested (3D MC-harv) and 2D monolayer-harvested (2D MNL-harv) cells. Cells expanded on 2D gelatin-coated monolayer cultures and enzymatically harvested (2D gelatin-MNL-harv) served as a control for the effects of gelatin coating during expansion, as the Cytodex 3 microcarriers are gelatin-coated.

**Fig. 3:**
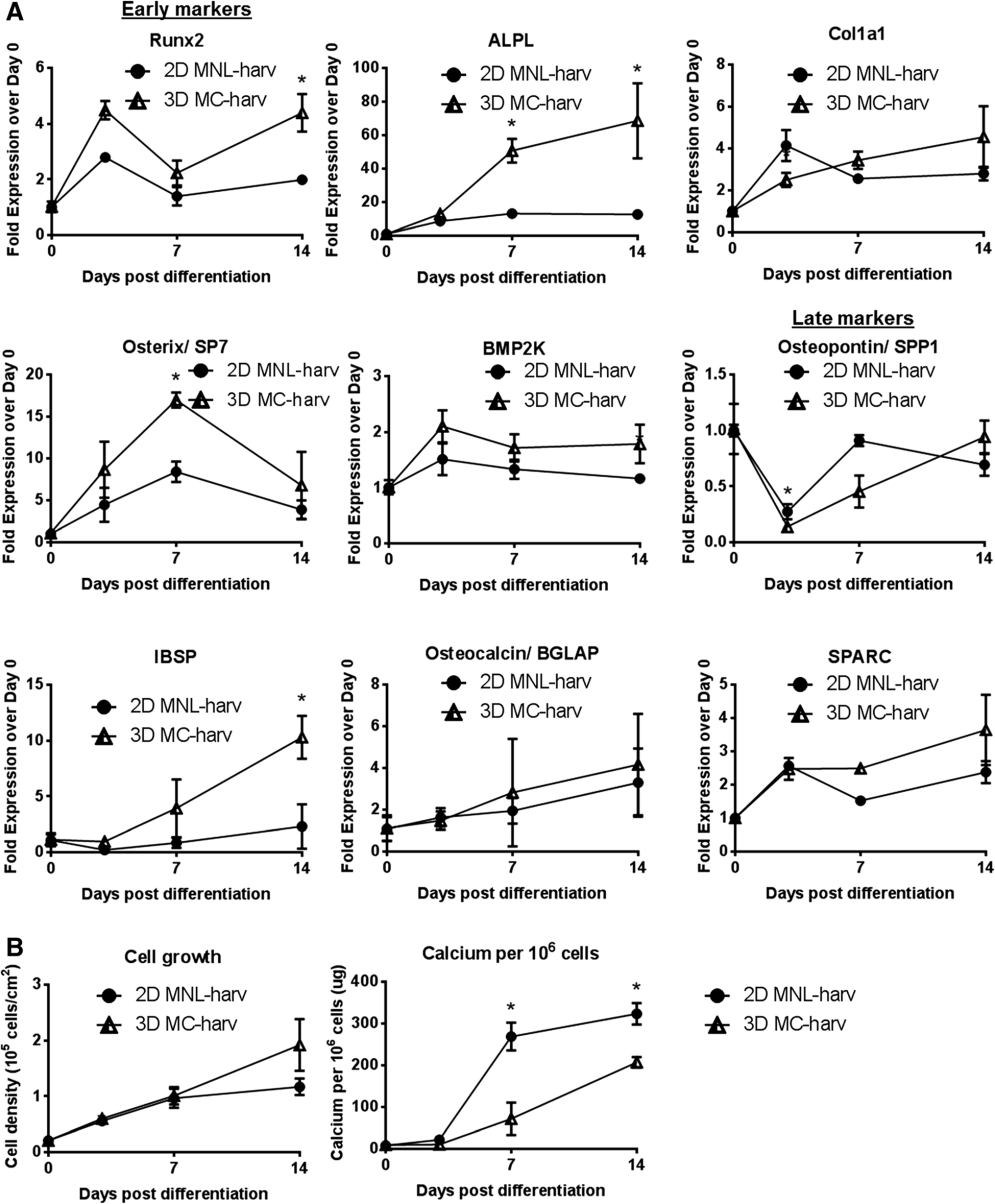
Kinetics of gene expression, cell growth and calcium deposition during osteogenic differentiation of collagen I-coated plates seeded with microcarrier-harvested (3D MC-harv) or 2D monolayer-harvested (2D MNL-harv) S27 hfMSCs. **a** Osteogenic gene expression, early and late markers. Values normalized to Day 0 post-differentiation (*p < 0.05 and >2-fold difference). *N* = 3 biological replicates per condition. **b** Cell growth (cell density in 10^5^ cells/cm^2^) (left) and calcium deposition per 10^6^ cells (right), (*p < 0.05). *N* = 3 biological replicates per condition. Two-way repeated measures ANOVA with post-hoc Tukey correction was performed between 3D MC-harv and 2D MNL-harv and MC-bound cells using Graphpad. Of the multiple comparisons performed, data representing a single comparison (2D MNL-harv vs 3D MC-harv) is shown in this figure

We examined early, and late osteogenic gene marker expression in hfMSCs undergoing osteogenic induction in order to study their differentiation potential. Cultures seeded with 3D MC-harv cells expressed higher levels of three early osteogenic markers, namely RUNX2, ALPL and Osterix/ SP7 and one late osteogenic marker, IBSP at a single time point (Fig. 3a). Despite the increased expression of these osteogenic markers, the mineralization of 3D MC-harv cells displayed slower kinetics compared to 2D MNL-harv cells. Specifically, 2D MNL-harv cells have 55 % higher calcium per 10^6^ cells at Day 14 compared to 3D MC-harv cells (Fig. 3b) and also stain for Alizarin red by Day 7, while 3D MC-harv cells do not stain at this timepoint (Additional file 2: Figure S2A). The control culture of 2D gelatin-MNL-harv hfMSCs did not display enhanced expression of early osteogenic genes (Runx2, ALPL and Col1a1) when compared with 2D MNL-harv hfMSCs (Additional file 3: Figure S1A). This suggests that the enhanced expression of early osteogenic genes in MC-harv hfMSCs (Fig. 3) is not due to the effects of the gelatin coating on Cytodex 3 microcarriers but rather to the mode of propagation (microcarriers in agitated spinner flask vs static MNL culture). Similar levels of COL1A1, BMP2K, Osteopontin/SPP1, Osteocalcin/BGLAP and SPARC gene expression level were observed in 3D MC-harv and 2D MNL-harv cells (Fig. 3a).

In agreement with results on the collagen-coated plates, on PCL-TCP scaffolds, 3D MC-harv cells also displayed increased expression of early (Runx2 and ALPL) but not late osteogenic markers compared to 2D MNL-harv cells (Additional file 4: Figure S3A). In contrast with observations on the collagen-coated plates, the decelerated mineralization of 3D MC-harv cells on 3D PCL-TCP scaffolds were not as apparent based on Alizarin Red staining compared to 2D MNL-harv cells (Additional file 2: Figure S2B).

### Effect of hfMSC harvesting on osteogenic differentiation (3D MC-bound vs 3D MC-harv cells)

To investigate the effect of cell harvesting on hfMSC osteogenic differentiation, we compared the kinetics of osteogenic gene expression and calcium mineralization during the differentiation process carried out in 6-well plates seeded with 3D MC-bound and 3D MC-harv hfMSCs.

**Fig. 4:**
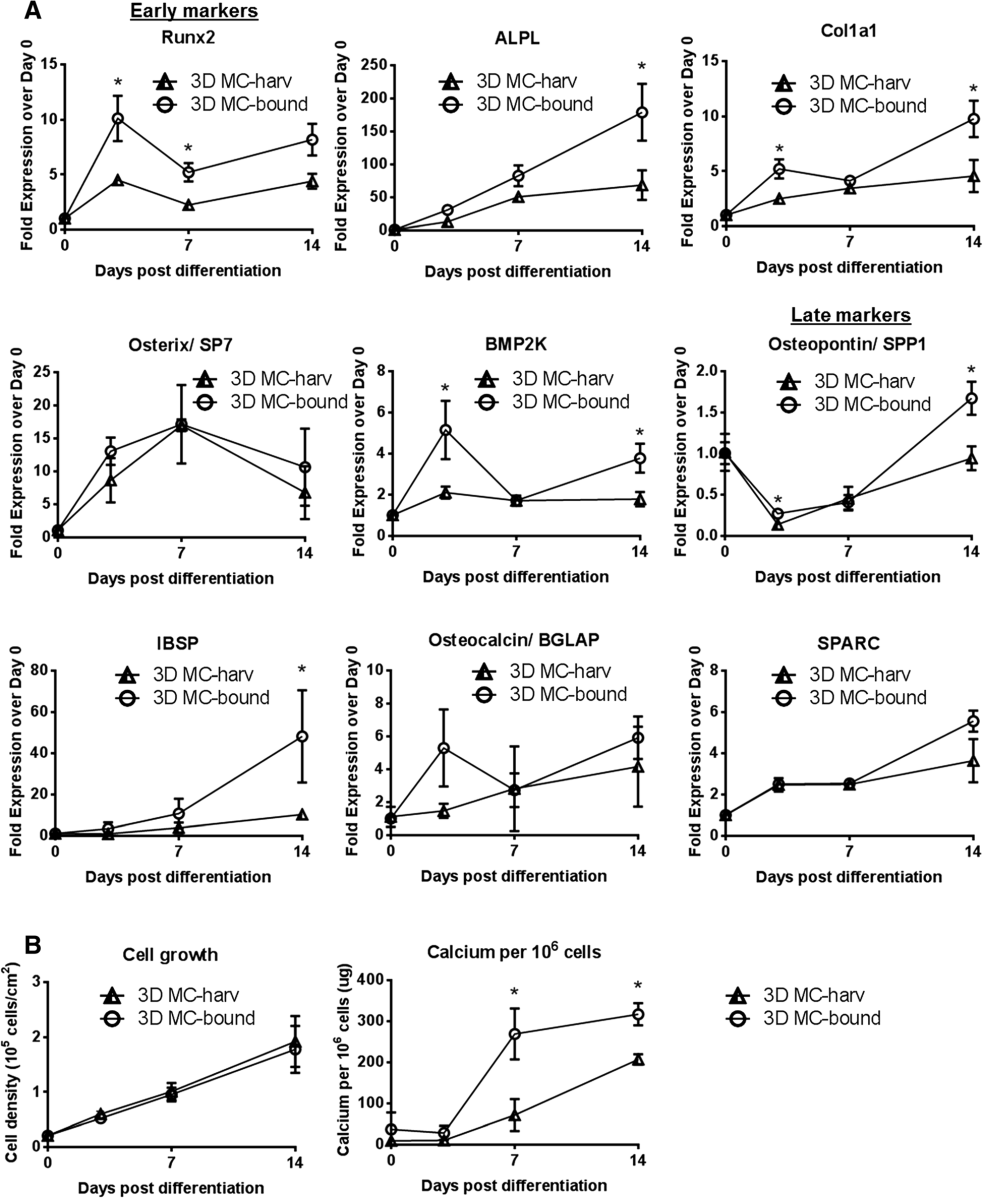
Kinetics of gene expression, early and late markers, cell growth and calcium deposition during osteogenic differentiation of collagen I-coated plates seeded with microcarrier-harvested (3D MC-harv) or microcarrier-bound (3D MC-bound) S27 hfMSCs. **a** Osteogenic gene expression values normalized to Day 0 post-differentiation (*p < 0.05 and >2-fold difference). *N* = 3 biological replicates per condition. **b** Cell growth (cell density in 10^5^ cells/cm^2^) (left) and calcium deposition per 10^6^ cells (right), (*p < 0.05). *N* = 3 biological replicates per condition. Two-way repeated measures ANOVA with post-hoc Tukey correction was performed between 3D MC-harv, 2D MNL-harv and 3D MC-bound cells using Graphpad. Of the multiple comparisons performed, data representing a single comparison (3D MC-bound vs 3D MC-harv) is shown in this figure

We found that cultures seeded with 3D MC-bound cells showed increased expression of early osteogenic markers such as RUNX2, ALPL, and COL1A1 as well as some late markers such as Osteopontin/ SPP1 and IBSP (Fig. 4a) compared to 3D MC-harv cells. Consistent with the increased expression of both early and late osteogenic genes, mineralization was accelerated in 3D MC-bound cells compared to 3D MC-harv cells (Fig. 4b). The calcium production rate was 1.5 to 3.8 fold higher in 3D MC-bound cell cultures as compared to 3D MC-harv cells. It is important to note that the calcium deposition occurred in foci surrounding the seeded 3D MC-bound cells as shown by the Alizarin red staining on day 7 and 14 (Additional file 2: Figure S2A) suggesting that hfMSCs attached to microcarriers have a higher mineralization potential.

### Comparison of efficiency of osteogenic differentiation (3D MC-bound vs 2D MNL-harv hfMSCs)

**Fig. 5:**
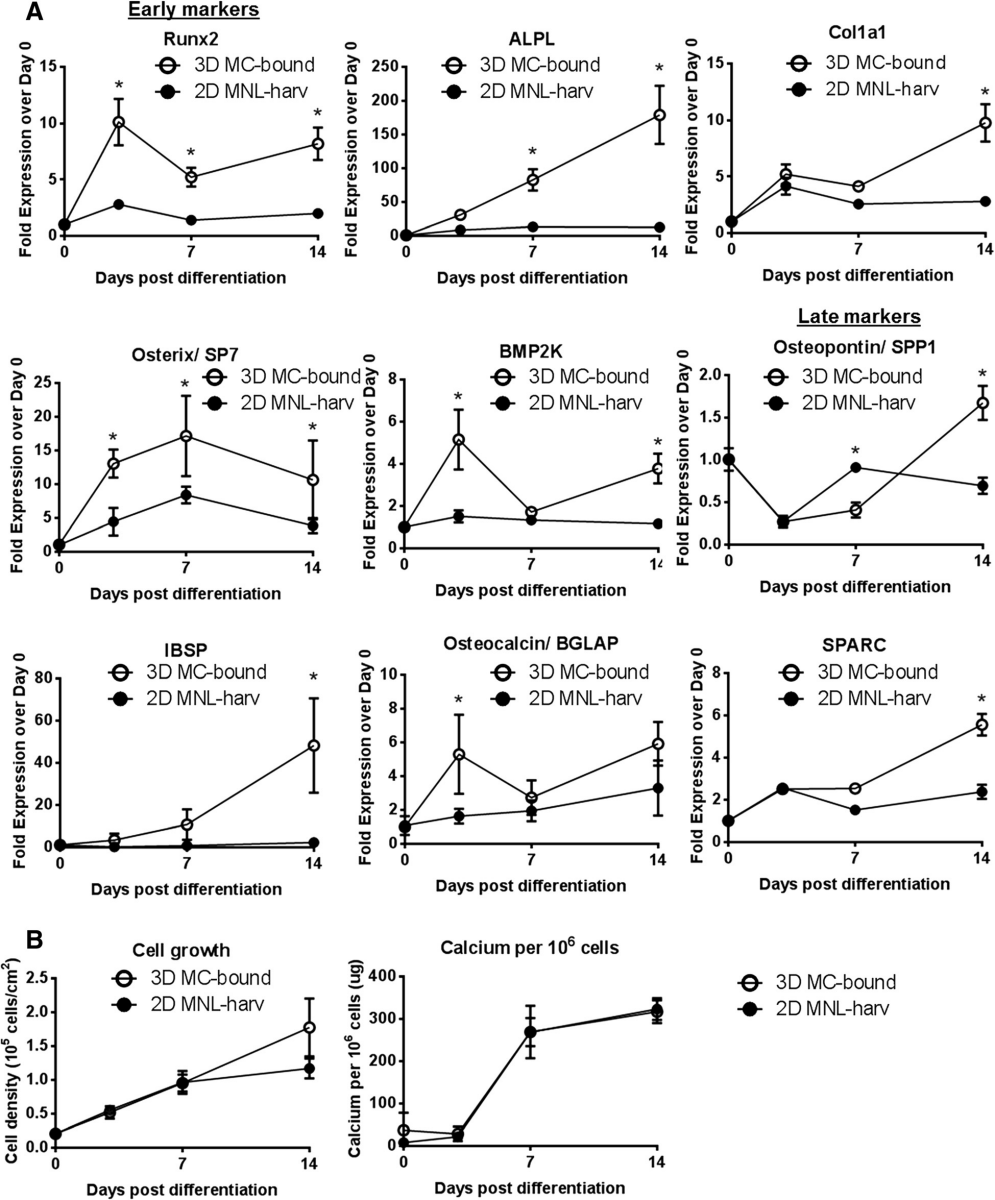
Kinetics of gene expression, early and late markers, cell growth and calcium deposition during osteogenic differentiation of collagen I-coated plates seeded with monolayer-harvested (2D MNL-harv) or microcarrier-bound (3D MC-bound) S27 hfMSCs. **a** Osteogenic gene expression values normalized to Day 0 post-differentiation (*p < 0.05 and >2-fold difference). *N* = 3 biological replicates per condition. **b** Cell growth (cell density in 10^5^ cells/ cm^2^) (left) and calcium deposition per 10^6^ cells (right), (*p < 0.05). *N* = 3 biological replicates per condition. Two-way repeated measures ANOVA with post-hoc Tukey correction was performed between 3D MC-harv, 2D MNL-harv and 3D MC-bound cells using Graphpad. Of the multiple comparisons performed, data representing a single comparison (3D MC-bound vs 2D MNL-harv) is shown in part (**a**) and (**b**) in this figure

Because the conventional method of *in vitro* expansion and MSC delivery involves cell culture on 2D tissue culture plastic monolayers (usually in cell stacks), we compared the osteogenic potential of 3D MC-bound cells to 2D MNL-harv cells. A control culture, 2D gelatin-MNL-harv hfMSCs, was added as previously discussed. 2D gelatin-MNL-harv hfMSCs did not show enhanced osteogenic gene expression or increased calcium deposition compared to either 3D MC-bound or 2D MNL-harv hfMSCs for 2 hfMSC cell lines, S27 and S127 (Additional file 3: Figure S1), showing that the gelatin coating during cell expansion do not affect osteogenic differentiation. In 3D MC-bound S27 cells differentiated on 6-well plates, gene expression levels of all 9 markers tested were elevated compared to 2D MNL-harv cells, in many cases at more than one time point (Fig. 5a). The genes that were upregulated in 3D MC-bound cells included early markers such as RUNX2, ALPL, COL1A1, Osterix/ SP7 and medium to late markers such as BMP2K, Osteopontin/SPP1, IBSP, Osteocalcin/BGLAP and SPARC (Fig. 5a). Although osteogenic gene expression levels were higher in 3D MC-bound cells during differentiation, for the S27 line, calcium deposition levels were equivalent to 2D MNL-harv cells as measured by calcium assay (Fig. 5b) and qualitative Alizarin Red staining (Additional file 2: Figure S2A).

When S27 hfMSCs were osteogenically induced on PCL-TCP scaffolds, 3D MC-bound S27 hfMSCs similarly expressed higher levels of RUNX2, ALPL, COL1A1, however there was no difference in the expression levels of Osteopontin/SPP1 and Osteocalcin/ BGLAP (Additional file 4: Figure S3B). While mineralization on collagen plates was merely equivalent between 3D MC-bound and 2D MNL-harv cells, on PCL-TCP scaffolds, 3D MC-bound cells displayed accelerated mineralization compared to 2D MNL-harv cells (Additional file 2: Figure S2B).

In order to confirm the biological significance of our findings, we repeated our experiment with a second hfMSC line known as S127. 3D MC-bound S127 hfMSCs showed upregulated gene expression of both early (Runx2, ALPL, Osteocalcin/BGLAP) and late markers (SPARC) (Additional file 3: Figure S1A) compared to 2D MNL-harv cells, confirming the result that was seen with 3D MC-bound S27 hfMSCs. Furthermore, 3D MC-bound S127 hfMSCs showed improved calcium deposition per million cells compared to 2D MNL-harv (Additional file 3: Figure S1B).

## Discussion

Fetal MSCs are may be a suitable MSC source for bone healing applications, as they have greater osteogenic capacity than umbilical cord- and adipose tissue-derived MSCs [3], and support in vivo bone formation [3–5]. The expansion of MSCs on microcarriers in bioreactors provides several advantages over current monolayer culture methods. These include scalability of culture in a cost effective manner [9], and the potential ability to deliver cells post-expansion without a cell harvesting step on biodegradable microcarriers [11, 12] for selected applications such as cartilage and bone healing. However, the effects of MSC culture in microcarrier/bioreactor systems and subsequent harvesting on cell phenotype and differentiation potential is not well understood.

hfMSCs cultured on 3D microcarriers versus 2D monolayer displayed a higher specific production rate of the pro- and anti-inflammatory and immunoregulatory [30] cytokine IL-6 [31] which maintains stemness in MSCs [32], the pro-angiogenic chemokine IL-8 [33, 34] and CXCL5, a chemokine which attracts leukocytes as well as hematopoietic stem cell migration[35]. These results suggest that the use of microcarrier-expanded hfMSCs in immunoregulatory or angiogenic therapies may provide greater benefits than with standard 2D MNL-harv cells. However, further experiments will be required to establish if this is the case with *in vitro* potency assays.

The high expression levels of ISCT MSC markers in hfMSCs expanded in both stirred 3D MC and 2D MNL cultures indicate that the mode of expansion did not alter the MSC-like phenotype of the hfMSCs. However, we observed a downregulation of CD146, an endothelial and pericyte marker, in 3D MC-expanded cells, and this effect also occurs in MSCs in spheroid culture [36], suggesting that the decrease in CD146 expression may be a response to the suspended nature of the cell culture.

Osteogenically induced cultures seeded with 3D MC-harv hfMSCs showed increased expression of early osteogenic genes and decelerated mineralization kinetics compared to 2D MNL-harv hfMSCs. While the reasons for this effect is unknown, possible factors involved may include differences in the cell microenvironment during expansion including substrate stiffness, shear forces due to agitation culture, or adhesion to a curved versus flat surface.

3D MC-bound hfMSCs showed improved osteogenic differentiation compared to both 3D MC-harv (Fig. 4, S2A) and 2D MNL-harv hfMSCs (Fig. 5, Additional file 3: Figure S1A & B, Additional file 2: Figure S2A & B and Additional file 4: Figure S3B). 3D MC-bound show increased expression of some early (Fig. 4) osteogenic genes, as well as accelerated mineralization compared to 3D MC-harvested hfMSCs (Fig. 4b, Additional file 2: Figure S2A). However, our most important finding was the enhanced differentiation of 3D MC-bound hfMSCs compared to 2D MNL-harv hfMSCs. Gene expression of osteogenic markers was elevated in 3D MC-bound S27 and S127 hfMSCs compared to 2D MNL-harv cells (Fig. 5a, Additional file 3: Figure S1A). Furthermore, 3D MC-bound hfMSCs showed equivalent (S27) (Fig. 5b) or enhanced calcium deposition (S127) (Additional file 3: Figure S1B) compared to 2D MNL-harv cells, which represent the industry standard method of cell expansion. These results confirm previous work from our lab which demonstrate that microcarrier-expanded and microcarrier-bound hfMSC undergo efficient mineralization [10].

The improved differentiation of 3D MC-bound hfMSCs could be explained by a number of factors include the lack of disruption of ECM-integrin or cell-cell contacts, or the localized regions of high cell confluency on and around the microcarriers. In support of the first explanation, ECM-integrin [37, 38] interactions play an important role in directing cell fate and we observed large differences in integrin gene expression between the 3D MC-bound and 3D MC-harv cells (Additional file 1: Figure S4). However, we also note that for 3D MC-bound hfMSC samples, Alizarin Red staining was higher on the cells attached to the microcarriers than on the cells which migrated off the microcarriers and onto the collagen-coated plate surface (Additional file 2: Figure S2A), suggesting that some aspect of the cell-microcarrier interaction enhances mineralization, in support of the second explanation. Our findings of enhanced osteogenic differentiation on 3D MC-bound hfMSCs raise questions of mechanism. The upregulation of osteogenic genes in 3D MC-bound hfMSCs over both 2D MNL-harv and 2D gelatin-MNL-harv hfMSCs, combined with the lack of enhancement in differentiation between gelatin coated and uncoated MNL-harv cells suggest that the results seen in 3D MC-bound hfMSCs are not due to the gelatin coating on Cytodex 3 microcarriers.

If the same enhancement of osteogenic differentiation occurs on other types of biodegradable/ biocompatible microcarriers, the expansion and delivery of hfMSCs on these microcarriers may offer multiple advantages over current methods of MSC expansion in 2D monolayer and harvesting prior to in vivo delivery for selected applications such as bone tissue engineering. These advantages include cost-efficient and large-scale production of hfMSCs in controlled bioreactors, as well as the potential elimination of the cell harvested process for select in vivo applications, enabling faster and lower cost MSC bioprocessing.

## Conclusion

This study indicates that hfMSCs which are cultured on 3D microcarriers in agitation culture and remain attached to Cytodex 3 gelatin-coated dextran microcarriers (3D MC-bound) during osteogenic induction show enhanced osteogenic differentiation compared to hfMSCs which are cultured under static conditions on 2D monolayer surfaces and are harvested prior to differentiation (2D MNL-harv). If these results also occur in biodegradable/biocompatible microcarriers, the method of microcarrier-expansion and in vivo delivery of 3D MC-bound hfMSCs may be a promising strategy which should be further evaluated for in vivo bone healing.

## Methods

### hfMSC expansion in spinner flasks and tissue culture plastic flasks

Fetal bone marrow derived MSCs were obtained from Dr Jerry Chan, NUS. These cells were harvested from fetal tissue with the approval of the institutional domain specific review board (NHG DSRB 2006/00154) and international guidelines regarding the use of fetal tissue for research purposes as previously described [5]. Pregnant women gave written consent for the procedure and the use of fetal tissue for research purposes. All fetal tissues were collected from fetuses after clinically indicated termination of pregnancy. Fetal bone marrow derived MSCs from two different donors were used in this study and these MSCs are referred to as S27 and S127. Cytodex 3 microcarriers (MCs), GE Healthcare, were prepared according to manufacturer’s instructions. Briefly, MCs were washed with PBS, autoclaved and rinsed in culture media prior to hfMSC seeding. P6-7 hfMSCs were seeded on either Cytodex3 MCs or T175 tissue culture plastic flasks. 8 mg/ml Cytodex3 MCs were seeded in 100 ml spinner flasks (Scientific Industries) at a density of 4 cells/microcarrier and stirred at 25 rpm overnight. Thereafter, the impeller rotation speed was increased to 30 rpm. hfMSCs were seeded at a density of 1142cells/cm^2^ in T175 tissue culture plastic flasks and cultured under static (no agitation) conditions. α-MEM medium supplemented with 10 % FBS, 1 % penicillin-streptomycin was used. Microcarrier cultures were fed by 50 % media change because it is technically challenging to perform full media changes without removing settled microcarriers. Full media changes were performed for plastic monolayer cultures every 2–3 days according to standard practice [10]. Cells were harvested after 7 days of expansion by enzymatic harvesting from microcarriers, 44 ml of 0.25 % Trypsin-EDTA (Gibco) was added to 800 mg of Cytodex 3 microcarriers which have a total surface area of 2160 cm^2^ (0.02 ml trypsin/cm^2^ growth area). After 15 min of incubation, trypsin was quenched with fresh growth media, and the microcarriers were separated from the cell suspension using a 40um cell strainer (Greiner). Cells in T175 plastic monolayer were trypsinized by rinsing with 5 ml PBS followed by 5 min of incubation with 4 ml of 0.25 % Trypsin-EDTA (0.02 ml trypsin/cm^2^ surface area) and quenching with 8 mls of growth media. The viable cell count in the cell suspension was obtained using the Nucleocounter (Chemometec) according to the manufacturer’s instructions. The average doubling time of the cells during the 7 day (168 h) expansion was calculated using the following equation:

T_d_ = (t_2_-t_1_) × log(2)/log(c_2_/c_1_) or T_d_ (hours) = 168 h × log(2)/log(c_2_/c_1_)

where T_d_ is doubling time, t_2_ is the final timepoint, t_1_ is the initial timepoint, c_2_ is the final cell count and c_1_ is the initial cell count.

### Differentiation of expanded hfMSCs on collagen-coated plates or PCL-TCP scaffolds

Cells were obtained post-expansion for seeding and differentiation in one of 3 ways (Fig. 1):

1. 3D microcarrier-expanded cells were not enzymatically harvested, but left attached to microcarriers and these cell-laden microcarriers were seeded (3D MC-bound)
2. 3D microcarrier-expanded cells were enzymatically harvested and seeded (3D MC-harv)
3. 2D uncoated monolayer tissue culture plastic flask-expanded cells were enzymatically harvested and seeded (2D MNL-harv)
4. 2D gelatin-coated monolayer plastic flask-expanded cells were enzymatically harvested and seeded (2D gelatin-MNL-harv)

Osteogenic differentiation was carried out in two systems:

1. 2D Collagen-coated 6-well plates
2. 3D PCL-TCP scaffolds

The 6-well plates were coated for 1 h at 37 °C with 0.01 % rat-tail collagen I (BD Biosciences). 6 well plates were then seeded with 200,000 hfMSCs per well. PCL-TCP scaffolds were obtained from Osteopore International were cut into 4 × 4 × 10 mm scaffolds and treated by rinsing in NaOH and washed three times in PBS. Scaffolds were seeded with 200,000 hfMSCs per scaffold.

After seeding, hfMSCs were cultured in osteogenic induction media as shown previously[10] (DMEM (Gibco) supplemented with 10 % FBS, 1 % penicillin-streptomycin, 10nM dexamethasone, 10 mM β-glycerophosphate and 0.2 mM ascorbic acid). We have used this osteogenic induction media formulation in previously published work with hfMSCs [10].

### Cell counting, surface marker flow cytometry

Cell counts of attached cells were performed by trypsinization of cells on 6-well plates or PCL-TCP scaffolds followed by nuclei counting according to the manufacturer’s instructions using the Nucleocounter NC-100, Chemometec. Cell counts were performed from 3 separate 6-wells within the same experiment (*n* = 3) for each condition tested. For flow cytometry, cells were trypsinized, resuspended in flow cytometry buffer (PBS/0.1 % BSA) and incubated with primary antibodies for 20 min, followed by incubation with a FITC-conjugated polyclonal rabbit anti-mouse secondary antibody for 15 min and analyzed on a GUAVA easyCyte HT sampling flow cytometer, Merck Millipore. We used anti-human primary antibodies for CD34, CD73, CD146, CD29 (itgβ1), CD49e (itgα5), CD49b (itgα2), CD49a (itgα1), CD51/61(itgαvβ3) and CD49f (itgα6) (Biolegend), CD90 (Millipore), CD105 (Invitrogen). We used the manufacturer’s recommended antibodies used as isotype controls (either mouse IgG1, IgG2a or IgG2b, or rat IgG2a from Biolegend or mouse IgG1 from Millipore).

### Measurement of cytokine production

Cytokine concentration (IL-6, IL-8 and CXCL-5) in culture supernatants were measured at days 0, 3, 4, 5, 6 and 7 using ELISA kits from R&D systems (Minneapolis, MN) according to the manufacturer’s instructions. Supernatant was obtained from 1 spinner flasks or T-flasks per condition (*n* = 1). For each time point, calculations were made using the formula below:

$$ \mathrm{Specific}\ \mathrm{cytokine}\ \mathrm{production}\ \mathrm{rate}\ \mathrm{at}\ \mathrm{Day}\ \mathrm{n}\ \left(\mathrm{pg}/\mathrm{cell}/\mathrm{day}\right)=\frac{Cytokine\  conten{t}_{{}_{Day\ n}}- Cytokine\  conten{t}_{{}_{Day\ n-1}}}{\left(\frac{Cell\  coun{t}_{{}_{Day\ n-1}} + Cell\  coun{t}_{{}_{Day\ n}}}{2}\right)\ } $$

### Quantitative RT-PCR

Total RNA was extracted from hfMSCs propagated in 6-well plates or scaffds by Trizol-chloroform extraction. RNA was extracted from 3 separate 6-wells within the same experiment (*n* = 3) for each condition tested. The RNA was purified using an RNeasy Mini kit (Qiagen) with on-column DNA digestion according to the manufacturer’s instructions. 0.5ug of total RNA was used to perform cDNA synthesis in 20ul total volume using a Maxima First Strand cDNA synthesis kit (Thermo Scientific). Quantitative PCR was carried out using Taqman Gene Expression Assays (Life Technologies) using the gene expression assays listed below. GAPDH was used as the housekeeping gene. The ID numbers for specific genes assayed using the Taqman Gene Expression Assay system are listed in Additional file 5: Table S1.

### Calcium deposition assay

Cells cultured on 6-well plates were rinsed with PBS three times and incubated with 0.5 N acetic acid for 1 h at room temperature to allow for calcium elution. Calcium was eluted from cells from 3 different wells per condition tested within the same experiment (*n* = 3). The calcium concentrations in the acetic acid samples were quantified using a calcium assay kit (BioAssay System) according to the manufacturer’s instructions. Final calcium concentration was determined after background subtraction of Day 0 values.

### Alizarin red staining

Differentiating cultures (in 6-well plates or scaffolds) were fixed with 4 % paraformaldehyde for 1 h at room temperature. Thereafter, cells were stained with 1 ml of 2 % alizarin red (Sigma), pH 4.2 solution for 1 h at room temperature, rinsed with PBS three times and imaged using an Evos light microscope. 3 different wells were stained per condition and a representative image is shown for each condition.

### Statistical analysis

Data are presented as mean and standard error. Statistical analysis was performed by one-way ANOVA with post-hoc Tukey (for data at a single time point) or two-way repeated measures ANOVA with post-hoc Tukey (for time course data) to compare between more than two groups. A *p*-value of less than 0.05 was considered significant. For gene expression data, a difference between two groups was considered significant if the *p*-value was less than 0.05 and there was a 2-fold or greater difference between the mean values for those groups. Statistical analysis was performed using Graphpad Prism software.

## Additional files

Additional file 1: Figure S4. Gene expression of anti-inflammatory, Wnt inhibition, YAP/TAZ targets and integrins S27 hfMSCs after 7 days of expansion in 2D monolayer-harvested cultures (2D MNL-harv) and harvested and non-harvested 3D MC cultures (3D MC-bound and 3D MC-harv respectively). Results are presented as fold expression levels of genes compared to 2D MNL-harv (**p* < 0.05 and at least a 2-fold difference in the means compared to 2D MNL-harv, ($ *p* < 0.05 and at least a 2-fold difference in the means compared to 3D MC-harv). (TIFF 609 kb) Additional file 2: Figure S2. Calcium deposition measured by Alizarin red staining during osteogenic differentiation of cultures seeded with 2D MNL-harvested, 3D MC-harv and 3D MC-bound S27 hfMSCs. (A) Collagen-coated 6-well plates. *N* = 3 biological replicates per condition, one representative image is shown per condition. (B) PCL-TCP scaffolds, (left) side view, (right) top view of scaffolds. *N* = 3 biological replicates per condition, one representative image is shown per condition. (TIFF 1729 kb) Additional file 3: Figure S1. Gene expression measured by qPCR and normalized calcium deposition at Day 14 post-osteogenic induction for 3D MC-bound, 2D MNL-harv and 2D gelatin-MNL-harv hfMSCs. (A) Osteogenic gene expression for S127 hfMSCs (**p* < 0.05 and >2-fold difference vs 2D MNL-harv, $ *p* < 0.05 and >2-fold difference vs 2D gelatin-MNL-harv,). *N* = 3 biological replicates per condition. (B) Calcium deposition per million cells for S27 and S127 hfMSCs (**p* < 0.05). Comparison between MC-bound to the 2 controls MNL-harv and gelatin-MNL-harv. For both (A) and (B) ANOVA with post-hoc Tukey was performed using Graphpad. (TIFF 303 kb) Additional file 4: Figure S3. Kinetics of osteogenic gene expression during osteogenic differentiation of S27 hfMSC in PCL-TCP scaffold cultures. (A) Cultures seeded with 2D monolayer-harvested (2D MNL-harv) and microcarrier-harvested (3D MC-harv) cells, (B) Cultures seeded with microcarrier-harvested (2D MNL-harv) and microcarrier-bound (3D MC-bound) cells. Two-way repeated measures ANOVA with post-hoc Tukey correction was performed between 3D MC-harv, 2D MNL-harv and 3D MC-bound cells using Graphpad, (**p* < 0.05 and at least a 2-fold difference in the means). Of the multiple comparisons performed, data representing a single comparison is shown: (A) 3D MC-harv vs 2D MNL-harv, (B) 3D MC-bound vs 2D MNL-harv. (TIFF 544 kb) Additional file 5: Table S1. List of Taqman Gene Expression Assay IDs for genes investigated by qPCR in this study. (DOCX 13 kb)

## Abbreviations

MSC: Mesenchymal stem cells or multipotent stromal cells
PBS: Phosphate buffered saline
3D: Three dimensional
2D: Two dimensional
MC: Microcarrier
MNL: Monolayer
PCL: Polycaprolactone
TCP: Tricalcium phosphate
PLGA: Poly lactic co-glycolic acid
PEG: Polyethylene glycol
PLA: Poly lactic acid
MEM: Minimal essential medium
EDTA: Ethylenediaminetetraacetic acid
