# Supplementary figures and images for: Enhanced in vitro osteogenic differentiation of human fetal MSCs attached to 3D microcarriers versus harvested from 2D monolayers
Source: BMC Biotechnol. 2015 Oct 31;15:102. doi: 10.1186/s12896-015-0219-8 (PMC4628389; doi:10.1186/s12896-015-0219-8)

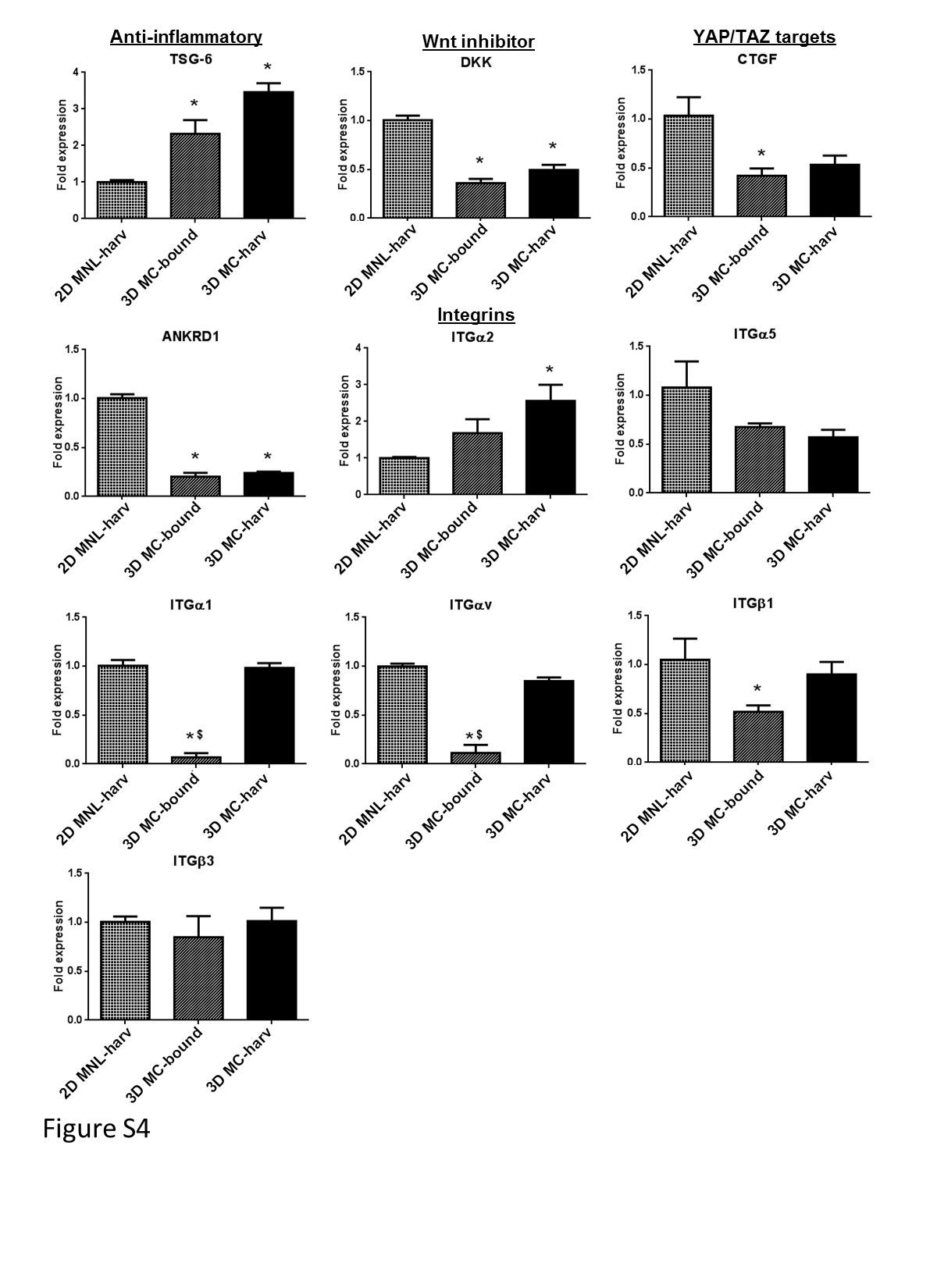

Supplement: Additional file 1: Figure S4. — Gene expression of anti-inflammatory, Wnt inhibition, YAP/TAZ targets and integrins S27 hfMSCs after 7 days of expansion in 2D monolayer-harvested cultures (2D MNL-harv) and harvested and non-harvested 3D MC cultures (3D MC-bound and 3D MC-harv respectively). Results are presented as fold expression levels of genes compared to 2D MNL-harv (*p < 0.05 and at least a 2-fold difference in the means compared to 2D MNL-harv, ($ p < 0.05 and at least a 2-fold difference in the means compared to 3D MC-harv). (TIFF 609 kb) [file 12896_2015_219_MOESM1_ESM.tif]

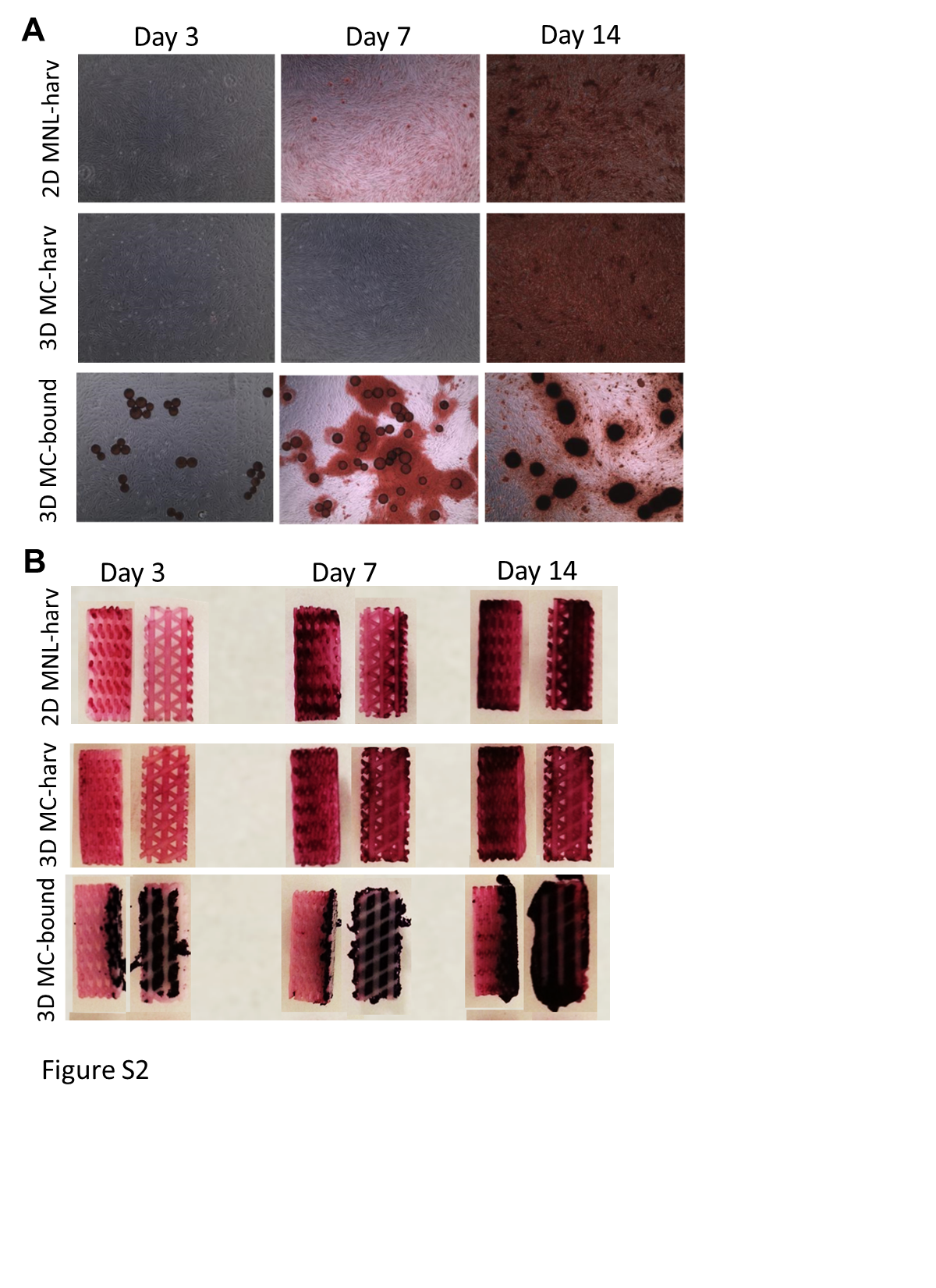

Supplement: Additional file 2: Figure S2. — Calcium deposition measured by Alizarin red staining during osteogenic differentiation of cultures seeded with 2D MNL-harvested, 3D MC-harv and 3D MC-bound S27 hfMSCs. (A) Collagen-coated 6-well plates. N = 3 biological replicates per condition, one representative image is shown per condition. (B) PCL-TCP scaffolds, (left) side view, (right) top view of scaffolds. N = 3 biological replicates per condition, one representative image is shown per condition. (TIFF 1729 kb) [file 12896_2015_219_MOESM2_ESM.tif]

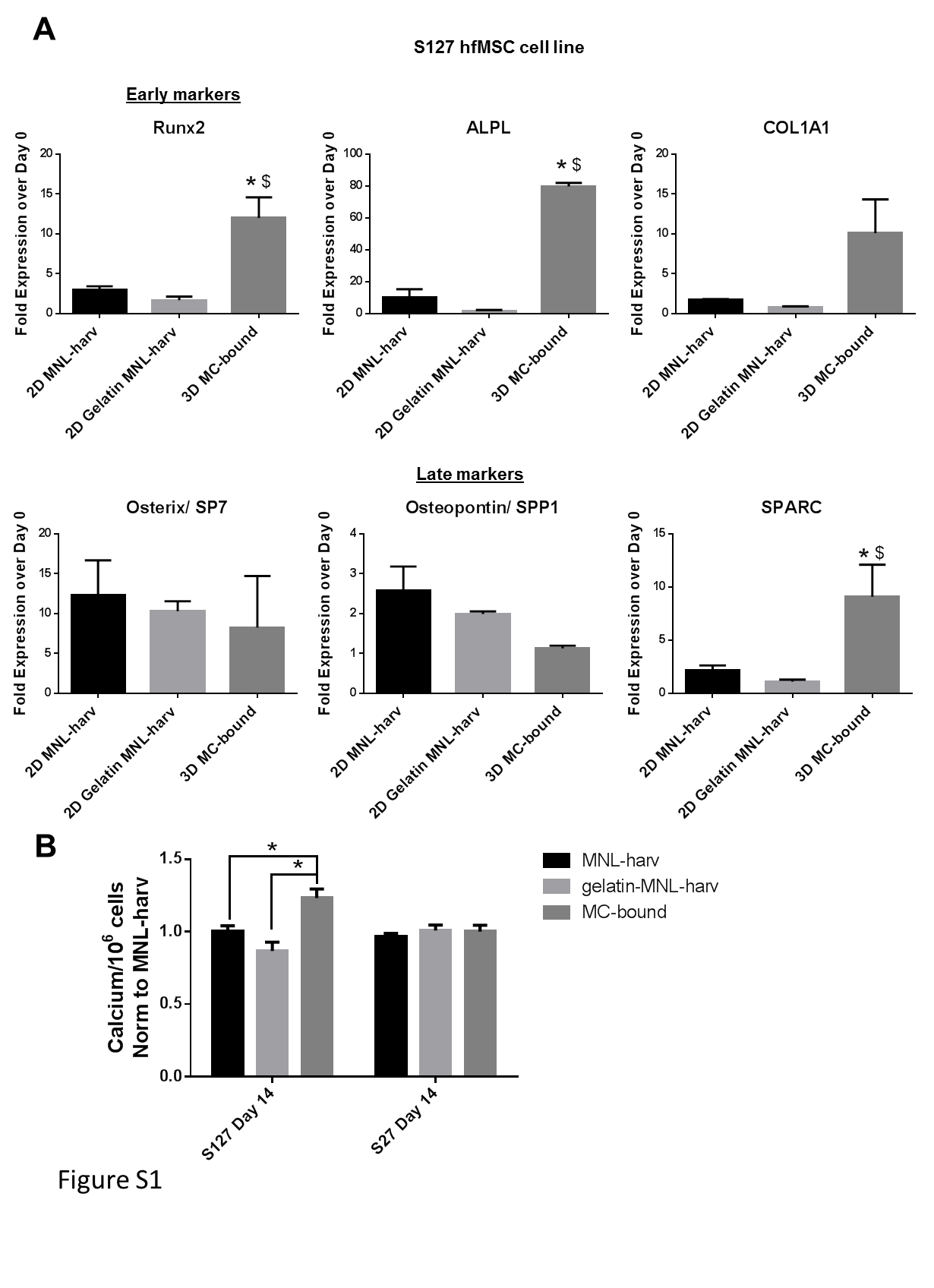

Supplement: Additional file 3: Figure S1. — Gene expression measured by qPCR and normalized calcium deposition at Day 14 post-osteogenic induction for 3D MC-bound, 2D MNL-harv and 2D gelatin-MNL-harv hfMSCs. (A) Osteogenic gene expression for S127 hfMSCs (*p < 0.05 and >2-fold difference vs 2D MNL-harv, $ p < 0.05 and >2-fold difference vs 2D gelatin-MNL-harv,). N = 3 biological replicates per condition. (B) Calcium deposition per million cells for S27 and S127 hfMSCs (*p < 0.05). Comparison between MC-bound to the 2 controls MNL-harv and gelatin-MNL-harv. For both (A) and (B) ANOVA with post-hoc Tukey was performed using Graphpad. (TIFF 303 kb) [file 12896_2015_219_MOESM3_ESM.tif]

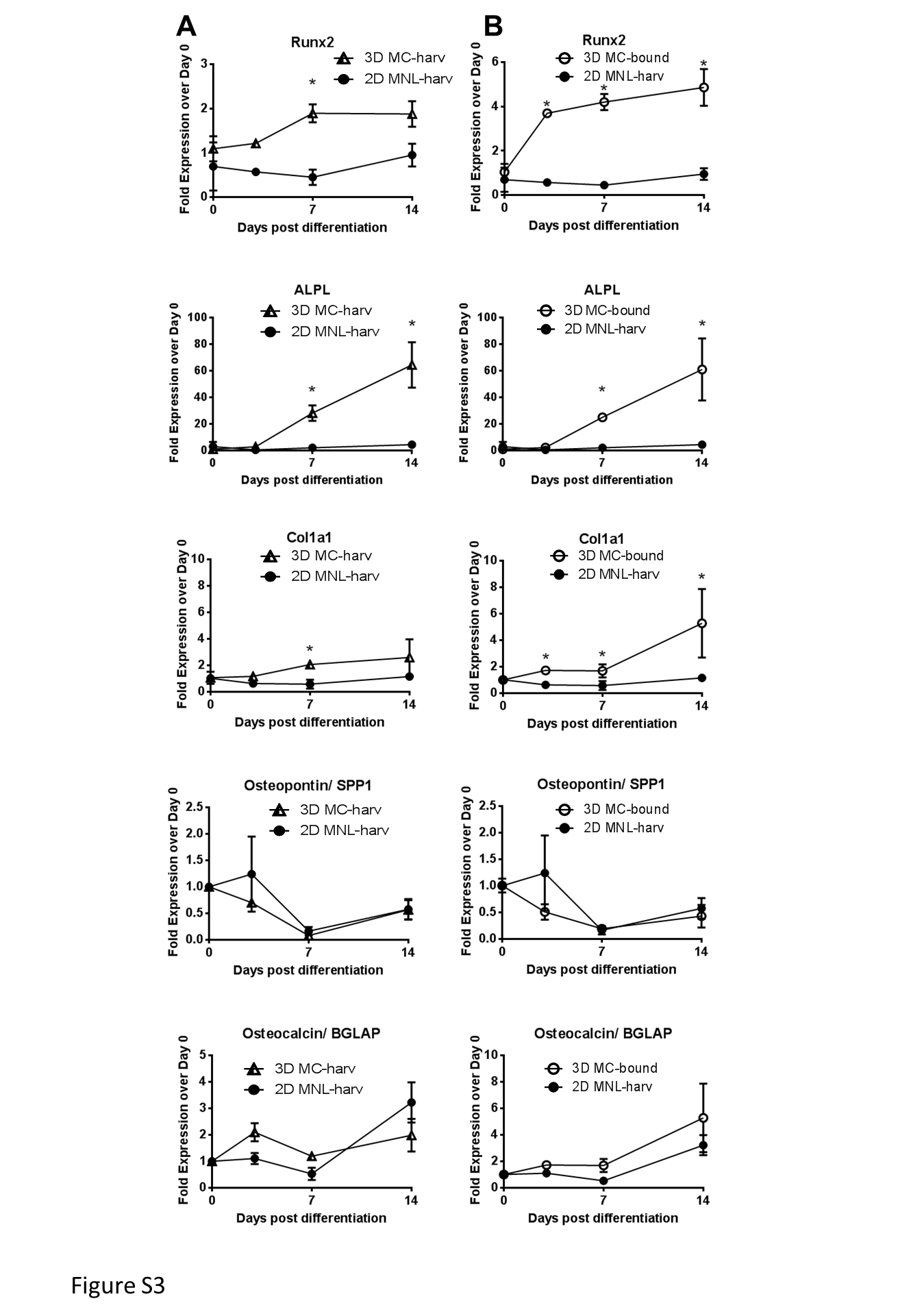

Supplement: Additional file 4: Figure S3. — Kinetics of osteogenic gene expression during osteogenic differentiation of S27 hfMSC in PCL-TCP scaffold cultures. (A) Cultures seeded with 2D monolayer-harvested (2D MNL-harv) and microcarrier-harvested (3D MC-harv) cells, (B) Cultures seeded with microcarrier-harvested (2D MNL-harv) and microcarrier-bound (3D MC-bound) cells. Two-way repeated measures ANOVA with post-hoc Tukey correction was performed between 3D MC-harv, 2D MNL-harv and 3D MC-bound cells using Graphpad, (*p < 0.05 and at least a 2-fold difference in the means). Of the multiple comparisons performed, data representing a single comparison is shown: (A) 3D MC-harv vs 2D MNL-harv, (B) 3D MC-bound vs 2D MNL-harv. (TIFF 544 kb) [file 12896_2015_219_MOESM4_ESM.tif]
