# Supplementary material for: Enhanced in vitro osteogenic differentiation of human fetal MSCs attached to 3D microcarriers versus harvested from 2D monolayers
Source: BMC Biotechnol. 2015 Oct 31;15:102. doi: 10.1186/s12896-015-0219-8 (PMC4628389; doi:10.1186/s12896-015-0219-8)
Supplement: Additional file 5: Table S1. — List of Taqman Gene Expression Assay IDs for genes investigated by qPCR in this study. (DOCX 13 kb) [file 12896_2015_219_MOESM5_ESM.docx]

| **Gene** | **Taqman Gene Expression Assay ID** |
| --- | --- |
| Osteocalcin/ BGLAP | Hs01587814_g1 |
| RUNX2 | Hs00231692_m1 |
| Osteopontin/ SPP1 | Hs00959010_m1 |
| ALPL | Hs01029144_m1 |
| IBSP | Hs00173720_m1 |
| SPARC | Hs00234160_m1 |
| BMP2K | Hs00214079_m1 |
| Osterix/ SP7 | Hs01866874_s1 |
| COLA1 | Hs00164004_m1 |
| ITGA2 | Hs00158127_m1 |
| ITGA5 | Hs01547673_m1 |
| ITGB1 | Hs00559595_m1 |
| ITGB3 | Hs01001469_m1 |
| ITGA6 | Hs01041011_m1 |
| TSG-6 | Hs01113602_m1 |
| DKK1 | Hs00183740_m1 |
| CTGF | Hs01026927_g1 |
| ANKRD | Hs00923599_m1 |
| GAPDH | Hs02758991_g1 |

Table S1
